# Depression and anxiety as barriers to art initiation, retention in care, and treatment outcomes in KwaZulu-Natal, South Africa

**DOI:** 10.1016/j.eclinm.2020.100621

**Published:** 2021-01-07

**Authors:** Michael Truong, Madhura S. Rane, Sabina Govere, Sean R. Galagan, Mahomed-Yunus Moosa, Ann Vander Stoep, Connie Celum, Paul K. Drain

**Affiliations:** a Department of Epidemiology, School of Public Health, University of Washington, 1959 Pacific St, Seattle, WA 98195, United States; b AIDS Healthcare Foundation, Durban, South Africa; c Department of Global Health, School of Public Health, University of Washington, Seattle, United States; d AIDS Healthcare Foundation, Durban, South Africa; e Department of Infectious Diseases, University of KwaZulu-Natal, Durban, South Africa; f Department of Medicine, School of Medicine, University of Washington, Seattle, United States

**Keywords:** Depression, Anxiety, Mental health, HIV, Antiretroviral therapy, South Africa

## Abstract

**Background:**

Since mental health may influence HIV care among people living with HIV (PLHIV), we sought to evaluate the impact of anxiety and depression on ART initiation and HIV-related outcomes.

**Methods:**

We conducted a prospective cohort study of PLHIV in the Umlazi Township of KwaZulu-Natal, South Africa. We measured depression using the Patient Health Questionnaire (PHQ-9) and anxiety using the Generalized Anxiety Disorder (GAD-7) scale, both of which have been validated in sub-Saharan Africa, among all patients prior to receiving a positive HIV test. We then followed those who tested HIV+ for 12 months to determine their time to ART initiation, missing clinic visits or refills, retention in care, hospitalization, and death. We used logistic regression models, adjusted for socio-demographic characteristics such as age and sex, to examine the effects of baseline measures of depression and anxiety on ART initiation and HIV treatment outcomes.

**Findings:**

Among 2,319 adult PLHIV, mean age was 33 years (SD=9.3 years), 57% were female, and baseline median CD4 count was 317 cells/mm^3^ (IQR=175–491 cells/mm^3^). In univariate analyses, depression was associated with slower rates of ART initiation. In adjusted models, PLHIV with depression had lower odds of initiating ART within 90 days of HIV testing (aOR=0.60, 95% CI=0.46, 0.79, *p*<0.01), and lower odds of being retained in care (aOR=0.77, 95% CI=0.60, 0.99, *p* = 0.04). By the end of the 12-month study period, odds of ART initiation among PLHIV with depression were higher than the first 90 days but still significantly lower compared to those without depression (aOR=0.72, 95% CI=0.52, 0.99, *p* = 0.04). Among PLHIV who initiated ART, depression was associated with a lower odds of missing clinic visits (aOR=0.54, 95% CI= 0.40, 0.73, *p*<0.01). Anxiety was strongly correlated with depression (*r* = 0.77, *p*<0.01) and had similar effects on HIV-related outcomes.

**Interpretations:**

The presence of depression is a significant barrier to ART initiation and retention in care among adult PLHIV in South Africa. Mental health screenings around the time of HIV testing may help improve linkage and HIV-related outcomes.

**Funding:**

This work was supported by the Infectious Disease Society of America Education & Research Foundation and National Foundation for Infectious Diseases (PKD); Massachusetts General Hospital Executive Committee on Research (PKD); the Harvard University Center for AIDS Research [AI060354] (PKD); and the National Institute of Allergy and Infectious Diseases [AI108293, AI143351] (PKD). The content is solely the responsibility of the authors and does not represent the official views of the National Institutes of Health or other funding agencies.

**Research in Context**

**Evidence before this study**

Prior to this research, authors conducted a search of existing literature on these topics utilizing; databases: PubMed, Web of Science, Semantic Scholar, Common keywords: depression, anxiety, mental health, HIV, ART, Sub-Saharan Africa, South Africa, (Search period: Sep 2017-August 2018)

**Added value of this study**

Research in the United States show an association between greater depression being associated with poor linkage to care, however this association has been understudied in sub-Saharan Africa. There is a paucity of research exploring the impact of mental health on HIV care initiation in South Africa, despite the evidence that PLHIV are at much higher risk of having poor mental health. Results from our study show that engagement in care is a challenge among PLHIV in South Africa with depression and further emphasizes the need to improve patient engagement and retention in care in order to achieve the 90–90–90 goals.

**Implications of all the available evidence**

Our results suggest that mental health disorders, such depression and anxiety, are significant barriers among PLHIV initiating ART and achieving good treatment outcomes in South Africa. Reducing delays in early linkage to care could be supported by offering mental health screenings alongside HIV testing to identify PLHIV with poor mental health, encouraging same-day initiation of ART, and increasing follow-up and counselling efforts for those people who need additional support. Mental health screenings around the time of HIV testing may help improve linkage and retention to HIV care and may be a critical intervention to achieving the 90–90–90 goals in South Africa.

Alt-text: Unlabelled box

## 1 Introduction

The Joint United Nations Programme on HIV/AIDS (UNAIDS) proposed the 90–90–90 goal that aims to have 73% of persons living with HIV (PLHIV) achieve viral suppression by the year 2020.^1^ This target describes the goal of having 90% of PLHIV being aware of their status, 90% of those diagnosed with HIV enrolled in antiretroviral therapy (ART), and 90% of those enrolled in ART achieving viral suppression. Despite recommendations for universal ART, regardless of CD4 T-cell count, many PLHIV in low- and middle-income countries (LMICs) have not initiated ART.^2^ South Africa has been making progress towards the 90–90–90 goal, and according to recent data has achieved 85–71–86.^3^ Although more PLHIV are becoming aware of their status (85%) and those receiving ART are achieving viral suppression (86%), ART initiation rates (71%) in South Africa still need to improve.

Some sociodemographic factors, such as younger age, being male, living in an urban district, and being employed have been found to be associated with poor linkage to HIV care in South Africa (initiating care within 30 days of HIV testing).^4^ However, there is a paucity of research exploring the impact of mental health on HIV care initiation in South Africa, despite the evidence that PLHIV are at much higher risk of having poor mental health.^5^ Depression and anxiety have already been found to be associated with some HIV-related outcomes, such as declining adherence,^6^^,^^7^ increases in viral load,^8^^,^^9^ increased risk of suicide,^10^ and increased mortality risk.^10^ Depression has also been associated with lower patient activation, with depressed individuals being less likely to initiate or engage in care than those without depression.^11^ Given the higher risk of poor mental health among PLHIV,^5^ mental health issues may present barriers for achieving the 90–90–90 goal by affecting engagement and retention in care in the era of universal test and treat.

Worldwide, there is limited research assessing the impact of mental health on HIV-related outcomes in LMICs, including South Africa. Therefore, we sought to better understand the role of mental health, specifically depression and anxiety, on the course of ART initiation, retention in care, and HIV treatment outcomes.

## 2 Methods

### 2.1 Site and participants

We conducted a prospective cohort study of HIV-positive adults recruited from the outpatient department of iThembalabantu Clinic in Umlazi Township, South Africa from September 2013 to April 2017. Eligible participants were 18 years of age or older, English or Zulu speaking, HIV seropositive and not receiving ART, and willing and able to provide written informed consent to participate in the study. We excluded those who were unable to give informed consent to participate in the study.

The study was approved by the University of Washington's Institutional Review Board (IRB #49,563) and the University of KwaZulu-Natal's Medical Research Ethics Committee (Protocol #BF052/13). All study participants provided written informed consent. This study manuscript adheres to the current Strengthening the Reporting of Observational Studies in Epidemiology (STROBE) guidelines.^12^

### 2.2 Data collection

Participants demographic information, clinical data, and biological specimens were collected for this study. Basic demographic information and baseline health-related information, including measurements for depression and anxiety, were collected from all potential participants before HIV testing, so as not to bias the respondent results from receiving HIV testing results. Patients who then tested positive for HIV were then enrolled into the study after research staff determined eligibility and obtained informed consent.

HIV testing was conducted by the clinic staff following standard of care procedures. Participants who tested positive for HIV were seen by a research nurse, who obtained vital signs and collected biological specimens, which included samples of blood, urine, and sputum. After the research nurse visit, all participants then proceeded with routine medical care for HIV, which includes testing to monitor CD4 T-cells and ART initiation according to South African guidelines.^13^ Research staff monitored participants’ course of treatment through medical records and conducted follow-up phone calls at three, six, and 12 months to schedule follow-up visits. All participants were followed for up to 14 months to determine HIV treatment outcomes and mortality status.

### 2.3 Measurement of exposures: depression and anxiety

We assessed depression using the PHQ-9 questionnaire,^14^ a 9-item questionnaire asking the participant about his or her depressive symptoms. This measure has been validated for use among populations in sub-Saharan Africa.^15^^,^^16^ For each item, responses were scored on a 4-point Likert scale (0 for “Not at all” through 3 for “Nearly every day”), with a total range from 0 to 27. The PHQ-9 has standardized categories reflecting levels of symptom severity: none (0–4), mild (5–9), moderate (10–14), moderately severe (15–19), and severe (20–27). We use the standardized PHQ-9 score ≥10 to define the presence of depression.

We assessed anxiety using the GAD-7,^17^ a 7-item questionnaire asking the participant about his or her anxiety symptoms. For each item, responses were scored on a 4-point Likert scale (0 for “Not at all” through 3 for “Nearly every day”), with a total range from 0 to 21. The GAD-7 also has standardized categories reflecting levels of symptom severity: none (0–4), mild (5–9), moderate (10–14), and severe (15–21). This measure has been validated for use among populations in sub-Saharan Africa.^18^ We use the standardized GAD-7 score ≥10 to define the presence of anxiety.

We assessed depression and anxiety status as binary indicator variables. Scores of ≥10 for the PHQ-9 and GAD-7 are recommended cutoff scores for assessing depression and anxiety in clinical settings, optimizing sensitivity and specificity. These cutoffs have been used for measuring mental health in sub-Saharan Africa.^19^^,^^20^

### 2.4 Outcome definitions

We recorded the date of ART initiation, and defined linkage to care as initiation of ART within 90 days of the date of HIV testing at the clinic. We defined retention in care as participants who were still actively receiving care (i.e., not yet lost to follow-up) within two months of the 12-month follow-up visit. Research staff recorded whether a participant had missed refilling their ART medications or missed any clinic visits at any point throughout the study period. We obtained data for follow-up CD4 T-cell count and HIV viral load test results closest to the 12-month end of study exit visit, and we determined change in these outcomes by comparing them to measured levels from the baseline visit. We accessed medical records from local hospitals, including the Prince Mshiyeni Memorial Hospital in Umlazi and the King Edward VIII Hospital at the University of KwaZulu-Natal, to record hospitalization events and death. We also matched a participant's South African ID number with the South African death registry to assess mortality.

We reviewed each participant's medical chart, including hospitalization, and attempted at least up to three calls to participants. Every hospitalized patient had an additional hospital chart review to determine the cause of hospitalization, including cryptococcal meningitis. For those whose vital status could not be obtained by the end of the study, we searched the South African national death registry for date of death. All participants were categorized as either retaining in care at the study clinic, transferred to another HIV clinic, lost to clinical follow-up, or deceased.

### 2.5 Statistical analyses

We used tests for independent samples (t-tests and chi-square tests) to examine associations between cohort demographic characteristics and outcomes. We measured the correlation between depression and anxiety using Pearson's correlation coefficient. We used univariate regression models to examine associations between depression and anxiety with outcomes of interest. We used logistic models to analyze binary outcomes (linkage to care, retention in care, missed refills, missed visits, lost to follow-up, ART initiation, hospitalizations and death), and linear models to analyze continuous outcomes (change in CD4 cell count and latest viral load, log-transformed). For each outcome, patients missing any outcome data were excluded from that analysis. We adjusted all multivariable models for age and sex, as they are considered common confounders for each of our outcomes, and conducted analyses in R (version 3.4.3) through the RStudio interface (version 1.1.419).^21^

### 2.6 Role of the funding source

The funder of the study (US National Institutes of Health) had no role in the study design, data collection, data analyses, results interpretation, or writing of the report.

## 3 Results

### 3.1 Cohort characteristics

Among 6385 participants enrolled, 2379 (37.3%) were HIV-positive and followed for up to 14 months. Among PLHIV, we excluded participants who were missing data on PHQ and GAD scores (*n* = 60), with the final cohort consisting of 2319 participants (Table 1). The average age of study participants was 33.1 years (standard deviation=9.3), and 1330 (57.4%) were female. Less than half were employed (43.2%), and the majority earned less than 2000 ZAR (South African rand) per month (75.7%), and about half (45.5%) had a university level education. The median CD4 T-cell count at baseline was 317 cells/mm^3^ (IQR=175–491 cells/mm^3^). The overall prevalence of depression was 12.5% (*N* = 291) and prevalence of anxiety was 9.3% (*N* = 216). Depression and anxiety were found to be strongly correlated (*r* = 0.77, *p* <0.01).

**Table 1:** Cohort characteristics at enrollment (*N* = 2319).

Cohort characteristic	N (%)
Age, mean (SD)	33.1 (9.3)
Sex	
Female	1330 (57.4)
Male	989 (42.6)
Education	
None (primary not completed)	454 (19.6)
Some high school (but not matric)	811 (35)
Higher degree (university)	1054 (45.5)
Employed (>20 h)	1002 (43.2)
Income <2000 ZAR (11.56 USD)/mo(*N* = 2297)	1738 (75.7)
Marital status (married)	145 (6.3)
Church attendance (*N* = 2135)	1689 (79.1)
BMI (*N* = 2317)	
Underweight (<18.5)	134 (5.8)
Normal (18.5–24.9)	1056 (45.6)
Overweight (25.0–29.9)	604 (26.1)
Obese (30+)	523 (22.6)
CD4 cell count at baseline, median (IQR)	317 (175, 491)
Depression	
Yes (PHQ=10–27)	291 (12.5)
No (PHQ=0–9)	2028 (87.5)
Anxiety	
Yes (GAD=10–21)	216 (9.3)
No (GAD=0–9)	2103 (90.7)

SD: standard deviation, ZAR: South African rand, BMI: body mass index, IQR: interquartile range, PHQ: Patient Health Questionnaire, GAD: Generalized Anxiety Disorder screening.

### 3.2 Risk factors for depression and anxiety

Participants with depression were, on average, older (35.4 years vs. 32.8 years, *p*<0.01), had higher education levels (*p*<0.0001) and higher income levels (*p* = 0.04) than those without depression. PLHIV with depression had higher prevalence of underweight BMI (13.1% vs. 4.7%, *p*<0.01) and a 67 cells/mm^3^ lower median CD4 T-cell count (vs. 262 cells/mm^3 vs.^ 329 cells/mm^3^, *p*<0.01) compared to those without depression (Table 2).

**Table 2:** Cohort characteristics among PLHIV with and without depression or anxiety (*N* = 2319).

	Depression			Anxiety		
Characteristic, N (%)	No (*N* = 2028)	Yes (*N* = 291)	*p*	No (*N* = 2103)	Yes (*N* = 216)	*p*
Age, mean (SD)	32.8 (9.1)	35.4 (10.1)	**<0.01**	32.8 (9.2)	35.8 (10.2)	**<0.01**
Sex (female)						
Female	1158 (57.1)	172 (59.1)	0.55	1205 (57.3)	125 (57.9)	0.92
Male	870 (42.9)	119 (40.9)		898 (47.0)	91 (42.1)	
Education						
None (primary not completed)	427 (21.1)	27 (9.3)	**<0.01**	436 (20.7)	18 (8.3)	**<0.01**
Some high school (but not matric)	656 (32.3)	155 (53.3)		699 (33.2)	112 (51.9)	
Higher degree (university)	945 (46.6)	109 (37.5)		968 (46)	86 (39.8)	
Employed (>20 h)	862 (42.5)	140 (48.1)	0.08	904 (43)	98 (45.4)	0.54
Income < 2000 ZAR (11.56 USD)/mo (*N* = 2297)	1537 (75.8)	201 (69.1)	**0.04**	1595 (75.8)	143 (66.2)	**0.01**
Marital status (married)	127 (6.3)	18 (6.2)	0.97	125 (5.9)	20 (9.3)	0.11
Church attendance (*N* = 2135)	1517 (74.8)	172 (59.1)	**0.02**	1559 (74.1)	130 (60.2)	0.78
BMI (*N* = 2317)						
Underweight (<18.5)	96 (4.7)	38 (13.1)	**<0.01**	106 (5.0)	28 (13.0)	**<0.01**
Normal (18.5–24.9)	925 (45.6)	131 (45.0)		948 (45.1)	108 (50.0)	
Overweight (25.0–29.9)	538 (26.5)	66 (22.7)		561 (26.7)	43 (19.9)	
Obese (30+)	468 (23.1)	55 (18.9)		487 (23.2)	36 (16.7)	
CD4 cell count at baseline, median (IQR)	328.5 (185, 502)	262 (132.5, 410.5)	**<0.01**	327 (182,502)	251 (130, 387)	**<0.01**

T-test significance testing for age, BMI, and CD4 count; Chi-Square significance testing for sex, education, employment, income, and marital status.

SD: standard deviation, ZAR: South African rand, BMI: body mass index, IQR: interquartile range.

Compared to those with depression, PLHIV with anxiety had similar sociodemographic trends, as compared to those without anxiety. Participants with anxiety were, on average, older (35.8 years vs. 32.8, *p*<0.01), had higher education levels (*p*<0.01), and higher income levels (*p* = 0.01). PLIV with anxiety had a higher prevalence of underweight BMI (13% vs. 5%, *p*<0.01) and a 76 cells/mm^3^ lower median CD4 cell count (251 cells/mm^3^ vs. 327 cells/mm^3^, *p*<0.01) compared to those without anxiety (Table 2).

### 3.3 Depression and study outcomes

Depression was associated with linkage to care, missed visits, and CD4 decline (Table 3). In univariate analyses, depression was significantly associated with decreased linkage to care (OR=0.67, 95% CI=0.51, 0.87), ART initiation (HR=0.73, 95% CI=0.64, 0.84), and fewer missed clinic visits (OR=0.51, 95% CI=0.37, 0.68). PLHIV with and without depression both experienced a decrease in CD4 count during the study period (11.6 cells/mm^3^ and 51.4 cells/mm^3^ respectively), however this decline was even greater among depressed, with a difference of 39.8 cells/mm^3^ (beta=−39.8, 95% CI=−57.8, −21.8) (Table 4).

**Table 3:** Distribution of ART initiation and HIV-related outcome for those with and without depression, and those with and without anxiety.

Characteristic, N (%)	Total (*N* = 2319)	Depression			Anxiety		
		No (*N* = 2028)	Yes (*N* = 291)	*p*	No (*N* = 2103)	Yes (*N* = 216)	*p*
Linkage to care (ART initiation within 3 months)	1707 (73.6)	1514 (74.7)	193 (66.3)	**<0.01**	1553 (73.8)	154 (71.3)	0.46
ART Initiation by 12 months	1945 (83.9)	1709 (87.9)	236 (81.1)	0.19	1759 (83.6)	186 (86.1)	0.39
Missed refills (*N* = 1858)	414 (22.3)	372 (22.7)	42 (19.1)	0.25	378 (22.5)	36 (20.5)	0.31
Missed visits (*N* = 2312)	735 (31.8)	676 (33.4)	59 (20.3)	**<0.01**	686 (32.7)	49 (22.7)	**<0.01**
Retention in care at 12 months	1543 (66.6)	1362 (67.2)	181 (62.2)	0.1	1403 (66.7)	140 (64.8)	0.62
Lost to follow-up	455 (19.6)	395 (19.5)	60 (20.6)	0.7	416 (19.8)	39 (18.1)	0.6
CD4 count^1^, mean (SD)	−16.6 (102.3)	−11.6 (92.3)	−51.4 (150.9)	**<0.01**	−13.4 (95.8)	−48.6 (148.8)	**<0.01**
Viral load^2^ (end), mean (SD)	5.6 (2.2)	5.6 (2.2)	5.4 (1.8)	0.14	5.6 (2.2)	5.5 (1.9)	0.39
Hospitalization or death	172 (7.4)	145 (7.1)	27 (9.3)	0.23	152 (7.2)	20 (9.3)	0.34

T-test significance testing for CD4 count and viral load; Chi-Square significance testing for linkage to care, ART initiation, missed refills and visits, retention in care, lost to follow-up, and hospitalization or death.

ART: antiretroviral therapy, SD: standard deviation.

1 Change from baseline to last CD4 reading.

2 Log-transformed viral load.

**Table 4:** Associations between mental health status and study outcomes.

	Depression				Anxiety			
	Univariate		Multivariate^1^		Univariate		Multivariate^1^	
	OR (CI)	*p*	aOR (CI)	*p*	OR (CI)	*p*	aOR (CI)	*p*
Linkage to care	0.67 (0.51, 0.87)	**<0.01**	0.6 (0.46, 0.79)	**<0.01**	0.88 (0.64, 1.20)	0.41	0.79 (0.58, 1.08)	0.14
ART initiation	0.8 (0.58, 1.10)	0.17	0.72 (0.52, 0.99)	**0.04**	1.21 (0.81, 1.81)	0.34	1.09 (0.72, 1.64)	0.68
Missed refills	0.8 (0.56, 1.15)	0.22	0.85 (0.59, 1.23)	0.39	0.89 (0.6, 1.13)	0.54	0.96 (0.65, 1.41)	0.82
Missed visits	0.51 (0.37, 0.68)	**<0.01**	0.54 (0.4, 0.73)	**<0.01**	0.6 (0.43, 0.84)	**0.02**	0.65 (0.46, 0.91)	**0.01**
Retention in care	0.8 (0.62, 1.04)	0.09	0.77 (0.6, 0.99)	**0.04**	0.92 (0.68, 1.23)	0.57	0.88 (0.65, 1.18)	0.38
Lost to follow up	1.07 (0.79, 1.46)	0.64	1.18 (0.87, 1.61)	0.28	0.89 (0.62, 1.28)	0.54	0.99 (0.68, 1.43)	0.95
Hospitalization or death	1.33 (0.86, 2.04)	0.18	1.19 (0.76, 1.85)	0.44	1.31 (0.8, 2.14)	0.27	1.15 (0.7, 1.89)	0.58
	**β (CI)**	***p***	**β (CI)**	***p***	**β (CI)**	***p***	**β (CI)**	***p***
CD4 count^2^	−39.8 (−57.83, −21.77)	**<0.01**	−39.64 (−57.61, −21.7)	**<0.01**	−35.27 (−55.8, −14.7)	**<0.01**	−35.05 (−55.6, −14.5)	**<0.01**
Viral load^3^	−0.17 (−0.4, 0.06)	0.14	−0.13 (−0.37, 0.1)	0.26	−0.12 (−0.38, 0.15)	0.39	−0.08 (−0.34, 0.19)	0.56

ART: antiretroviral therapy, CI: 95% confidence interval, aβ: adjusted beta coefficient, aOR: adjusted odds ratio, β: beta coefficient, OR: odds ratio.

1 Adjusted for age and sex.

2 Change from baseline to last CD4 reading.

3 Log-transformed viral load.

In a multivariate model adjusted for age and sex, depression was associated with 40% lower odds of being linked to care (adjusted odds ratio [aOR]=0.60, 95% CI= 0.46, 0.79) within 90 days of testing positive for HIV and 28% lower odds within the year (adjusted odds ratio [aOR]=0.72, 95% CI=0.52, 0.99).On average, depressed individuals had a greater decrease in CD4 T-count than those without depression in these multivariate analyses, with a difference of 39.6 cells/mm^3^. Among those who did initiate ART, multivariate models adjusting for age and sex showed depression to be associated with a lower odds of missing visits (aOR=0.54, 95% CI=0.40, 0.73) throughout the study but also a lower odds of being retained at the end of the study (aOR=0.77, 95% CI=0.60–0.99) (Table 4).

### 3.4 Anxiety and study outcomes

Anxiety was associated with missed visits and CD4 decline (Table 3). In univariate analyses, anxiety was significantly associated with lower odds of missing clinic visits (OR=0.60, 95% CI=0.43, 0.84). PLHIV with and without anxiety both experienced a decrease in CD4 the study period (13.4 cells/mm^3^ and 48.6 cells/mm^3^ respectively), however this decline was even greater among those with anxiety, with a difference of 35.27 cells/mm^3^ (beta=−35.27, 95% CI=−55.80,−14.70), which was similar to the change among PLHIV with depression (Table 4).

In multivariate analyses, PLHIV with anxiety had a greater decrease in CD4 T-count than those without anxiety, with an absolute CD4 T-cell difference of 35.1 cells/mm^3^. While depression was associated with lower odds of being linked to and retained in care, these effects were not present among PLHIV with anxiety (Table 4).

## 4 Discussion

In this study of adult PLHIV in a township of South Africa, the presence of depression or anxiety as measured prior to HIV testing was strongly associated with delayed ART initiation and poor HIV-related outcomes. Depressive and anxiety symptoms were both common and strongly correlated, but depression was more strongly associated with adverse HIV treatment outcomes. The prevalence of depression and anxiety observed in this study was comparable to other similar studies in the field.^22^^,^^23^ Presence of anxiety was associated with a greater decline in CD4 T-cell count (compared to those without anxiety) throughout the study period. However, in addition to greater CD4 cell decline, depression was associated with lower odds of linkage to care within 90 days, overall ART initiation within the study period, and retention in care. Although both depression and anxiety were associated with fewer missed clinic visits, depressive disorders appear to be an initial barrier for patients to engage in HIV care. Both depression and anxiety were not found to be associated with loss to follow-up, hospitalization, and death. Results from this study illustrate the negative outcomes associated with poor mental health among adult PLHIV and emphasize the importance of identifying these individuals early in their HIV care and treatment.

The effect of depression on ART initiation has been measured in some studies examining the role of mental health in HIV care-seeking behavior. Findings from previous research in the United States show an association between greater depression being associated with poor linkage to care,^24^ however this association has been understudied in sub-Saharan Africa and was confirmed in our study. Recent research on mental health among PLHIV in South Africa has shown depression to be associated with delayed care-seeking behavior such as HIV testing,^25^ and our current study extends prior study results by demonstrating the degree to which depression impacts the cascade of HIV care. ART initiation among PLHIV with depression was significantly lower not just within 90 days of receiving an HIV+ diagnosis, but it also remained significantly lower even throughout the 12-month study period. This may suggest that patients who do not engage in care early on may also be much less likely to seek in care later on, emphasizing the importance of actively reaching out to patients early and encouraging engagement in care. Results from our study show that this is a challenge among PLHIV in South Africa and further emphasizes the need to improve patient engagement and retention in care in order to achieve the 90–90–90 goals.

Since depression has been negatively correlated with patient activation,^11^ individuals who report depressive symptoms may be less likely to actively manage their own healthcare. Less motivation to initiate care may lead to worse HIV-related outcomes, such as even lower CD4 cell count over time compared to non-depressed PLHIV. This has been shown in other studies in other sub-Saharan countries,^26^ but has also been confirmed with our study results. In our cohort, depression was a key barrier to this goal and highlights the need to identify and treat PLHIV who experience poor mental health.

In our setting, depression was associated with a lower risk of missing clinical visits, which was an unexpected finding. Other studies conducted in sub-Saharan Africa have shown that treating depression improves ART adherence and clinic attendance.[27], [28], [29], [30] Thus, seeking care for depression could have potentially impacted engagement in HIV care through more frequent clinic visits. Although depressed PLHIV were less likely to miss visits throughout the study overall, they also had a lower likelihood of remaining engaged in care by the end of the study. This finding might suggest that although these patients might initially miss fewer visits after being linked to care, they can still struggle to be retained in care over a longer period. Ongoing care efforts are important in ensuring that not only do these individuals find care but also continue to remain actively engaged in their care throughout the entire course of treatment.

Our study had several strengths and limitations. A major strength of the study was measuring depression and anxiety before patients were tested for HIV, the potential bias of knowledge of a positive HIV diagnosis on mental health status was avoided. Additionally, the prospective cohort design enables us to establish the temporal sequence between patient mental health status and subsequent HIV-related outcomes. However, we did not routinely collect data on mental health referrals and treatment, so an accurate estimate of patients in our study population receiving mental health treatment could not be determined. Including measures such as these would help to further elucidate the relationships between mental health and HIV-related outcomes.

In conclusion, our results suggest that depression and anxiety, are significant barriers among PLHIV initiating ART and achieving good treatment outcomes in South Africa. Although these findings are specific to South Africa, they may also be relevant to other countries in sub-Saharan Africa with similar ART initiation and mental health challenges. Reducing delays in early linkage to care could be supported by offering mental health screenings alongside HIV testing to identify PLHIV with poor mental health, encouraging same-day initiation of ART, and increasing follow-up and counselling efforts for those people who need additional support. Mental health screenings around the time of HIV testing may help improve linkage and retention to HIV care and may be a critical intervention to end the AIDS epidemic.

Fig. 1

**Fig. 1:**
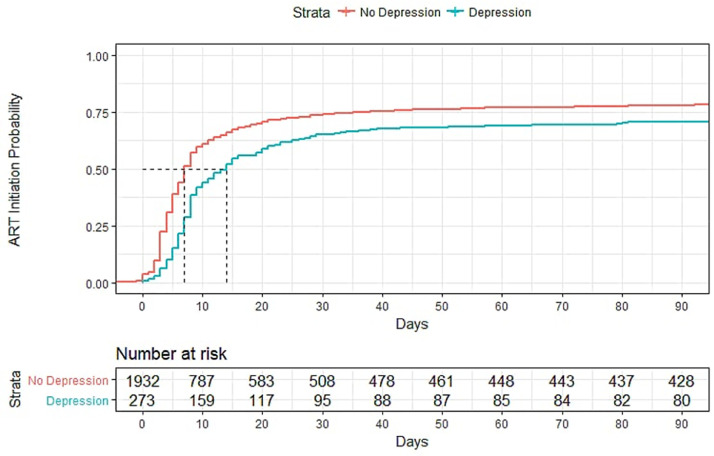

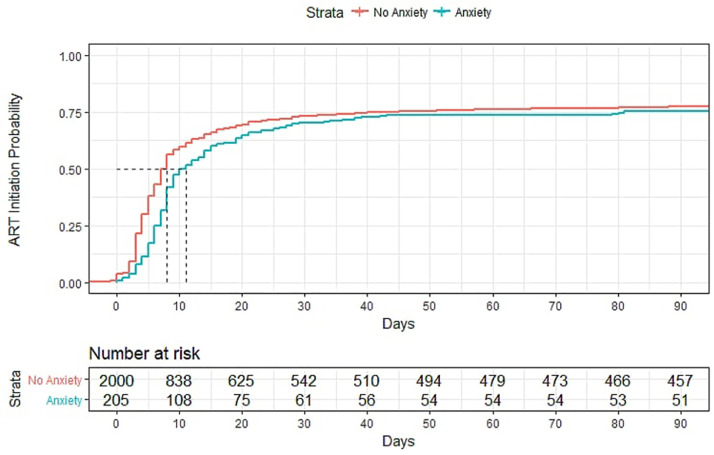
**(A)** Days for PLHIV to initiate ART by depression status over 90 days after HIV diagnosis. (**B)** Days for PLHIV to initiate ART by anxiety status over 90 days after HIV diagnosis.

## Declaration of Competing Interest

Dr. Drain reports receiving consulting or speaking fees from Gilead Sciences and Alveo Technologies, and research support from the NIH, CDC, Gilead Sciences, and the Bill and Melinda Gates Foundation. Dr. Celum reports grants from NIMH, during the conduct of the study; personal fees from Merck, personal fees from Gilead Sciences, outside the submitted work. We clare no other competing interests.

## Appendix Supplementary materials

Image, application 1

Image, application 2
