# Supplementary material for: Depression and anxiety as barriers to art initiation, retention in care, and treatment outcomes in KwaZulu-Natal, South Africa
Source: eClinicalMedicine. 2021 Jan 7;31:100621. doi: 10.1016/j.eclinm.2020.100621 (PMC7806795; doi:10.1016/j.eclinm.2020.100621)

**Figure 1A.** Days for PLHIV to initiate ART by depression status over 90 days after HIV diagnosis.


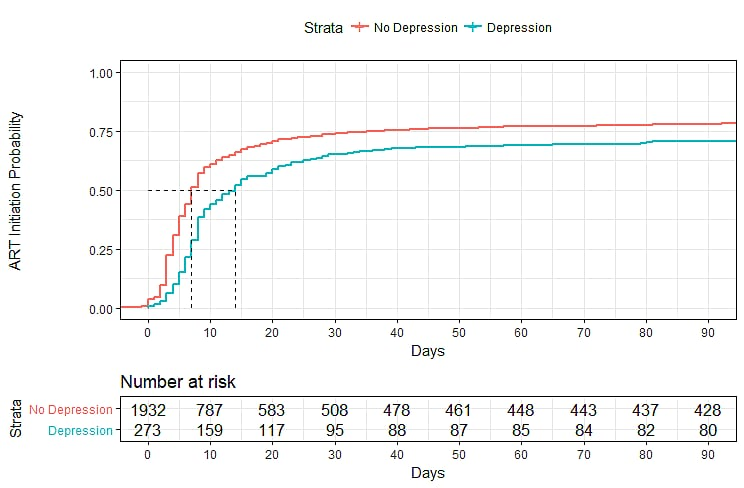


**Figure 1B.** Days for PLHIV to initiate ART by anxiety status over 90 days after HIV diagnosis.


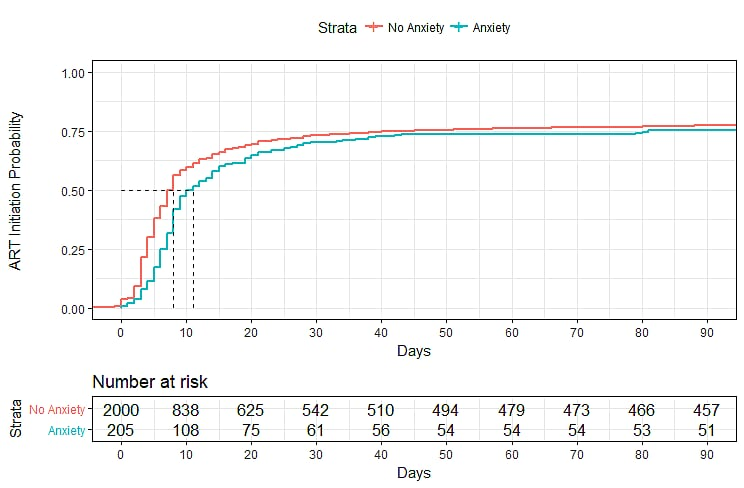

Supplement: Supplementary file 2 [file mmc2.docx]
